# Demographic, Laboratory, and Clinical Comparison of Pediatric Brucella Cases With and Without Liver Involvement

**DOI:** 10.7759/cureus.10862

**Published:** 2020-10-09

**Authors:** Mehmet Agin, Yusuf Kayar

**Affiliations:** 1 Pediatric Gastroenterology, Van Education and Research Hospital, Van, TUR; 2 Gastroenterology, Van Education and Research Hospital, Van, TUR

**Keywords:** brucellosis, elevated liver enzymes, hepatomegaly, children

## Abstract

Introduction

In this study, the purpose was to compare the demographic, clinical, and laboratory results of pediatric *Brucella* cases with and without liver involvement.

Methods

The data of 248 patients between 2 and 18 years of age at diagnosis with Brucellosis between July 2017 and August 2018 were analyzed retrospectively. Liver involvement was defined as elevated transaminase enzymes when compared to levels of the control group. Transaminases enzyme levels were taken as the control group. The two groups were compared in age, gender, complaints at admission, duration of symptoms, physical examination findings, laboratory values, blood culture reproduction, and relapse rates.

Results

There was no significant relationship between age and sex between groups with liver involvement (n = 92) and without liver involvement (n = 156). Loss of appetite, nausea, and sensitive stomach were higher in the patients who had hepatic involvement. In the patients who had hepatic involvement, the hemoglobin and platelet values ​​were lower, and the erythrocyte sedimentation rate (ESR), C-reactive protein (CRP), and blood culture growth were higher (p < 0.05). The relapse rates were lower in patients who had liver involvement (p < 0.05).

Conclusions

The correlation detected between blood culture positivity and elevated liver enzymes, CRP and ESR levels, low hemoglobin and platelet levels were considered to be consistent with the fact that brucellosis is a pathogen that involves the reticuloendothelial system.

## Introduction

Brucellosis is a disease seen frequently in many parts of the world, particularly in developing countries. It is originally a disease of animals and can be transmitted to humans during the slaughter or care of infected animals or the ingestion of contaminated meat and milk products. Brucellosis involves many organs, leading to a variety of complications, making diagnosis in humans difficult due to its subtle manifestations [1,2].

Brucellosis is seen endemically in the Arabian Peninsula, India, Mexico, Central and South America, and Mediterranean countries. It is estimated that there are 500,000 new cases of brucellosis in the world on an annual basis. According to the frequency order in Turkey, it is mostly seen in the Southeastern Anatolia, Central Anatolia, and Eastern Anatolia regions [1].

The majority of the cases become ill in three to four weeks after their exposure to active agents [3]. After the infection, the bacteria multiply in the regional lymph nodes and enter into the blood. The disease involves mostly the reticuloendothelial system, but it can also affect other organ symptoms and ultimately cause different clinical manifestation [4,5]. Since very different clinical findings may be detected at all ages, the diagnosis is difficult. If it is not treated in a timely and effective manner, chronicity, complications, and relapses may be faced [5,6].

During a *Brucella* infection, although the liver is involvement is almost present, the increase in elevated transaminases is usually at a minimal level. Impairments are detected in elevated transaminases in approximately 25% of patients who have acute or chronic brucellosis [7]. Liver and spleen involvements are also detected in approximately 30-60% of the cases who have brucellosis [8].

Brucellosis is an important and widespread infectious disease in the Eastern Anatolian region of Turkey especially in Van and its surroundings where husbandry is common. In this study, we aimed to evaluate and compare the demographic, clinical, and laboratory results of children who had brucellosis with liver involvement and without specific organ involvement.

## Materials and methods

The data of 248 patients who were between 2 and 18 years of age in the Eastern Anatolian region and who were diagnosed with brucellosis between July 2017 and August 2018 were analyzed retrospectively. Data analysis included demographic data, signs and symptoms, laboratory findings, physical findings, treatment regimens, and outcome. The patients whose onsets of symptoms were shorter than 8 weeks were evaluated as acute, those between 8-52 weeks were evaluated as subacute, and those that lasted more than 52 weeks were evaluated as chronic. The blood culture samples were studied using the BacT/Alert systems (Organon Teknica, Durham, NC, USA) for 21 days. The diagnosis of brucellosis was made based on positive* Brucella* standard agglutination test (STA) test results (titer > 160) in the presence of clinical signs and symptoms suggestive of brucellosis or a four-fold increase in the serum antibody concentration in a serum sample obtained at two- to three-week intervals and/or isolation of *Brucella* spp. from the blood, bone marrow, or any body fluid or tissue culture.

The patients were divided into two groups: those who had liver involvement and those who did not. Liver involvement was defined as follows: (a) serum alanine transaminase (ALT) > 40 U/L and serum aspartate aminotransferase (AST) > 40 U/L, (b) palpation of the liver under the ribs in physical examination, and (c) detecting hepatomegaly in abdominal ultrasonography. Granulomatous hepatitis, hepatic abscess, cholecystitis, and diffuse hepatitis were diagnosed by abdominal ultrasonography. Transaminases were evaluated as liver involvement. Transaminases levels that were normal were included in the control group. Elevated AST and ALT levels in patients with hepatomegaly were included in the liver involvement group. Patients with hepatomegaly in the absence of transaminases enzyme elevation were considered as the control group. In our study, we excluded the most common liver diseases including viral hepatitis, metabolic disorders, autoimmune liver diseases, and biliary and neoplastic diseases that can be associated with elevated liver enzymes.

The patients who had liver involvement and the patients in the control group were compared in terms of age, gender, complaints at admission, mean duration of symptoms, time of clinical diagnosis, history of drinking raw milk and eating fresh cheese, family history of dealing with animal husbandry, physical examination findings (hepatomegaly, splenomegaly, fever, abdominal tenderness, etc.), laboratory findings (leukocytes, platelets, hemoglobin, AST, ALT, alkaline phosphatase [ALP] gamma glutamyl transferase [GGT], bilirubins, albumin, total protein, C-reactive protein [CRP], erythrocyte sedimentation rate [ESR]), treatment response, relapse rate, and blood culture reproduction.

Trimethoprim-sulfamethoxazole (10 mg/kg/day), rifampicin (20 mg/kg/day), and gentamicin (5-7 mg/kg/day) combination was administered to children who were under the age of eight years. Doxycycline (4 mg/kg/day), rifampicin (20 mg/kg/day), and streptomycin (20 mg/kg/day) were administered to the children who were older than eight years. All patients received oral treatment for six weeks. Follow-up was recommended to the patients in the first, third, and sixth months, and at the first year after the treatment. The patients whose symptoms and signs continued after the treatment completion were evaluated as unresponsive to the treatment. Having similar complaints and findings at any period in one year after the end of the treatment, increase in Brucella SAT titer, Rivanol Brucella SAT result being > 1/160, and/or reproduction in blood culture were accepted as relapse. The groups with and without liver involvement were compared for relapse and time of clinical diagnosis.

Patient informed consent and ethics committee approval

Verbal and written informed consent was obtained from all the patients included in the study and from their parents. After the study was completed, the study result of each patient was reported to his/her own parents. Ethics committee approval for the study was given by Van Education and Research Hospital Clinical Research Ethics Committee, Van, Turkey.

Statistical analysis

The normality of distribution of continuous variables was tested using the Kolmogorov-Smirnov test. The Mann-Whitney U test was used to compare two independent groups for non-normal data. The chi-square test applied to investigate the relationship between two categorical variables. For numerical data, median [minimum-maximum] values and for categorical data frequencies and percentages were given as descriptive statistics. Statistical analysis was performed using SPSS for Windows Version 24.0 (IBM Corp., Armonk, NY, USA) and a p-value < 0.05 was accepted as statistically significant.

## Results

In the patients who had liver involvement (n = 92) and in the control group (n = 156), the mean age during the diagnosis was 9.5 ± 4.1 years and 10.4 ± 4.1 years, respectively; there were no statistically significant differences in this respect. No significant differences were detected between the two groups in terms of gender. In a total of 89% of the patients who had liver involvement, there was a history of raw milk and fresh cheese intake; 77% had a family history of animal husbandry and 30% had a family history in this respect. In the control group, on the other hand, there was a history raw milk intake and fresh cheese consumption in 92%, 79% dealt with animal husbandry, and 24% had a family history of brucellosis. No statistically significant differences were detected between the two groups. Although the growth rate of the pathogen was 35% (87/248) in the blood culture in all the brucellosis cases, it was 49% (45/92) in the cases that had liver involvement and 27% (42/158) in the control group, and statistically significant differences were detected between the groups (p < 0.05). The relapse rates were 15% (36/248) in all the brucellosis cases, 9% (8/92) in the cases that had liver involvement, and 18% (28/156) in the control group. Statistically, the relapse rate was determined to be lower at a significant level in the cases that had liver involvement (p < 0.05). Hepatomegaly was detected in 48% and splenomegaly was detected in 33% of the patients who had liver involvement, and hepatomegaly was detected in 13% and splenomegaly in 8% in the control group. There were statistically significant differences between the groups (p < 0.05) (Table 1).

**Table 1:** Comparison of the demographic data of the groups.

Variables	Liver involvement			p-Value
	Yes (N = 92)	No ( N = 156)	Total (N = 248)	
	Mean ± SD	Mean ± SD	Mean ± SD	
Age at diagnosis (years)	9.5 ± 4.1	10.4 ± 4.1	9.9 ± 3.9	0.194
Mean duration of symptoms (days)	21.6 ± 17.6	21.2 ± 17.1	21.5 ± 17.2	0.197
	N (%)	N (%)	N (%)	
Time of clinical diagnosis				
Acute	67 (73%)	111 (71%)	178 (72%)	0.917
Subacute	25 (27%)	44 (28%)	69 (28%)	
Chronic	-	-	-	
History raw milk and fresh cheese intake (yes)	82 (89%)	144 (92%)	226 (91%)	0.613
History of animal husbandry (yes)	71 (77%)	123 (79%)	194 (78%)	0.805
Family history (yes)	28 (30%)	38 (24%)	66 (27%)	0.285
Blood culture positivity	45 (49%)	42 (27%)	87 (35%)	0.001
Relapse	8 (9%)	28 (18%)	36 (15%)	0.046
Hepatomegaly	44 (48%)	20 (13%)	64 (26%)	0.001
Splenomegaly	30 (33%)	12 (8%)	42 (17%)	0.001

Symptoms and physical examination findings of the groups were compared. Loss of appetite, nausea, and sensitive stomach were significantly more frequent in patients that had liver involvement (p < 0.005) (Table 2).

**Table 2:** Comparison of symptoms and physical examination findings of the groups.

Symptom	Liver involvement			p-Value
	Yes (N = 92), N (%)	No (N = 156), N (%)	Total (N = 248), N (%)	
Fever	83 (90%)	140 (90%)	223 (90%)	0.949
Muscle and joint pain	75 (82%)	131 (84%)	202 (81%)	0.541
Weakness	40 (44%)	93 (60%)	133 (54%)	0.012
Abdominal pain	45 (50%)	76 (49%)	121 (49%)	0.950
Nausea	48 (52%)	29 (19%)	77 (31%)	0.001
Lack of appetite	40 (44%)	41 (26%)	81 (33%)	0.005
Sensitive stomach	59 (64%)	44 (28%)	103 (42%)	0.001

The mean hemoglobin and mean platelet counts were lower to a significant degree in the liver involvement group when compared to the control group, and no significant differences were detected in mean white blood cell counts. The AST, ALT, CRP, and ESR values were higher in patients who had liver involvement at a significant level compared to the controls; however, no significant differences were detected in terms of total bilirubin, direct bilirubin, albumin, total protein, ALP, and GGT (Table 3). Transaminase elevation was higher than 10 times the upper limit of normal in six cases and was 642 ± 167 I/U for AST on average and 791 ± 143 I/U for ALT on average. Elevated CRP levels were detected in 49% (121/248) of all the cases that had brucellosis, 59% (54/92) of the cases that had liver involvement, and 43% (67/156) of the control group. ESR elevation was detected in 46% (114/248) of all the brucellosis cases, n 77% (71/92) of the cases that had liver involvement, and 28% (43/156) of the control group, which was statistically significant (p < 0.05) (Table 3).

**Table 3:** Comparison of the laboratory values of the groups. AST, aspartate aminotransferase; ALT, alanine transaminase; ALP, alkaline phosphatase; GGT, gamma glutamyl transferase; CRP, C-reactive protein; ESR, erythrocyte sedimentation rate

Variables	Liver involvement		p-Value
	Yes	No	
	Median (min-max)	Median (min-max)	
Leucocyte count (range: 4.5-10 x 10^3^/µL)	6950 (2400-48000)	7000 (2600-27900)	0.954
Hemoglobin (range: 12-16 g/dL)	11.5 (8.5-15)	12.3 (4.4-14.6)	0.001
Platelets (range: 150-450 x 10^3^/µL)	256000 (7900-589000)	289000 (14000-569000)	0.001
AST (range: 0-40 IU/L)	77 (41-854)	25 (5-122)	0.001
ALT (range: 0-40 IU/L)	83 (43-995)	19 (7-86)	0.001
Total bilirubin (range: 0.6-1.2 mg/dL)	0.8 (0.4-4)	0.7 (0.4-4.2)	0.897
Direct bilirubin (range: 0.1-0.3 mg/dL)	0.4 (0.1-3)	0.3 (0.1-2.5)	0.911
Albumin (range: 3.5-5.5 g/dL)	3.6 (3.1-4.1)	3.7 (3.2-4.2)	0.960
ALP (range: 50-350 IU/L)	113 (100-136)	115 (105-146)	0.871
GGT (range: 0-60 IU/L)	58 (54-89)	66 (50-90)	0.891
CRP (range: 0-5 g/L)	26 (0.2-100)	2.6 (0.08-99)	0.001
ESR (range: 0-20 mm/h)	25 (3-80)	10 (2-56)	0.001

## Discussion

Brucellosis is a chronic zoonotic infection and is among the most widespread zoonoses globally [9]. It is still endemic, especially in the eastern regions of Turkey [10]. The disease has a wide variety of clinical manifestations. It has a well-known predilection for the reticuloendothelial system, which may manifest as hepatitis, hepatomegaly, splenomegaly, and peripheral lymphadenopathy [11]. There is no universal definition in determining the liver involvement in brucellosis. Depending on the study and the definition of liver involvement, the reported frequency of hepatic manifestations is variable. Yet, because the liver is the largest reticuloendothelial system organ in the human body, its involvement in brucellosis is common over a wide clinicopathological spectrum, from mild and transient transaminitis to a severe disease, such as hepatic abscess [12].

The most common route of transmission of brucellosis is the consumption of animal foods such as unpasteurized milk and cheese. In this study, it was observed that the majority of the patients lived in rural areas and consumed unpasteurized milk and cheese. Another route of transmission of the disease is contact with sick/contaminated animals, or animal secretions [13]. The fact that the patient population comes from rural areas may be related to the more frequent consumption of fresh cheese, and the possible lower sociocultural level in rural areas may cause insufficient measures to prevent disease. Having a history of brucellosis in the family of the individual can also be considered as a risk factor for the occurrence of the disease [14]. In 91% of our brucellosis cases, there was a history of consuming raw milk and raw cheese, and 78% of cases were of patients dealing with animal husbandry. No significant differences were detected between the patients who had and did not have liver involvement in terms of consuming raw milk and fresh cheese and a family history of dealing with animal husbandry.

It was reported in previous studies that consuming raw milk and fresh cheese was between 53% and 91% of cases [15-17]. Animal contact, or a family history of animal husbandry, was reported to be between 15% and 50% in previous studies [15-17]. In this study, similar rates were obtained in both groups. In previous studies conducted in our country or abroad, a family history of brucellosis was reported as being between 9% and 50.9% [18-20]. In studies conducted in Turkey, the range reported was 3-33% [15]. In our study, however, this rate was seen in 35% of the patients who had brucellosis, 30% in patients who had liver involvement, and 24% in the control group. This supports the arguments that brucellosis may be usually associated with milk and dairy products that are commonly consumed, and we believe that the history may be a diagnostic clue.

Sahinturk et al. showed that there was no significant relationship between liver involvement with age and gender [12]. Similarly, in our study, we found that there was no significant relationship between liver involvement with age and gender. The duration of symptoms is 14-28 days after ingestion of bacteria [13]. The mean duration of symptoms was reported to be 31.85 ± 44.3 days in the study of Yoldas et al. and four weeks in the study of Celebi et al. [14,21]. In our study, the first admission was approximately three weeks after the onset of complaints, and no significant correlation was found between duration of symptoms and liver involvement. The production of the agent in brucellosis patients ensures a definite diagnosis [3,4]. In various studies, the reproduction of the active agent in children who had brucellosis varied between 23.5% and 59.7% [2,15]. *Brucella* spp. reproduced in 35% of all brucellosis patients in our study. Furthermore, it was reproduced in 49% of the patients who had liver involvement and in 27% in the control group. Since the most reliable method for diagnosis is blood culture, blood culture must be sent for all cases that are suspected to have brucellosis. Positive blood culture was independently associated with liver involvement in patients with brucellosis, which can be interpreted as indicating the duration and severity of the disease.

As brucellosis can involve different organs and systems, it may present with different symptoms and findings. It was reported in previous studies that fever, weakness, loss of appetite, and nausea were the most common complaints of brucellosis at admission [14,22]. Similarly, fever, weakness, loss of appetite, and nausea were the most common complaints in all our cases that had brucellosis. In patients who had liver involvement, loss of appetite, nausea, and abdominal pain were more frequent. We think that the reason for the high rate of complaints in patients with liver involvement is excessive inflammation and the response of the immune system.

Laboratory research is typically unhelpful in the differential diagnosis of pediatric brucellosis because CRP, leukocyte count, and ESR are normal or slightly raised in most cases [14]. Typically, ESR, transaminases, and CRP are some of the most commonly used non-specific laboratory tests in diagnosis. One of the usage areas of ESR is the diagnosis and follow-up of infectious diseases [23]. Prior research has shown that ESR values to be between 49% and 72% [15-17]. CRP is one of the first acute phase reactants that heighten during inflammatory diseases and is used to assess the activity of the disease [23]. Previous studies have shown CRP to range between 40% and 72% [15,17]. In this study, ESR was identified in 46% of all brucellosis cases and in 77% of the cases with liver involvement, and was considerably greater in patients with hepatic involvement. In addition, CRP was detected in 49% of all patients suffering with brucellosis and in 59% of the cases that had liver involvement, and was this statistically significant in patients with hepatic involvement.

Hematological studies on the active course of brucellosis reported anemia in 44%, thrombocytopenia in 5%, and pancytopenia in 14% [24]. Furthermore, pancytopenia was detected in 10% of children inflicted with brucellosis [25]. The frequency of brucellosis with pancytopenia ranges from 3% to 21% in previous studies, where it is relatively greater in adults than in children [26]. The possible mechanisms suggested for pancytopenia include hypersplenism, an autoimmune process, granuloma formation in the bone marrow, bone marrow depression associated with septicemia, and phagocytosis of formed components by reticuloendothelial cells. This study demonstrates that platelet counts and hemoglobin were substantially lower in patients with liver involvement.

Diffuse hepatic involvement is usually reported during the course of brucellosis. Involvement of the liver varies, a slight increase in transaminase levels, mild hepatosplenomegaly, chronic suppurative disease, and, more rarely, acute hepatitis could be encountered [27]. Elevated transaminases were reported in 18-66% of pediatric cases [15,17], and, in our study, it was detected in 37% of all the brucellosis cases. Our data are consistent with the values that are reported in the literature. In addition, transaminases were found to be significantly higher in patients with liver involvement. Hepatomegaly was reported at a rate between 4.6% and 63% in previous studies [17,28,29]. In our study, too, it was detected in 26% of all the cases that had brucellosis, 48% of the cases that had liver involvement, and 13% in the control group, which is in accordance with the literature. In previous studies, splenomegaly was reported to be between 6.7% and 33% [15,17,18]. In our study, when all the cases with brucellosis were evaluated, splenomegaly was detected in 17% of all the cases, 33% of the cases that had liver involvement, and 8% in the control group. It was significantly higher in the group that had liver involvement. Hepatomegaly and splenomegaly vary according to the severity of the disease, whether it is chronic or not, and the presence of primary involvement in the related organs.

## Conclusions

The detection of the correlation between blood culture positivity and elevated liver enzymes, CRP and ESR levels, and low hemoglobin and platelet levels were considered to be consistent with the fact that brucellosis is a pathogen that involved the RES. Importance must be given to education and preventive studies, especially in endemic areas, because of the lack of specific clinical findings for childhood brucellosis, the occurrence of complications, loss of labor force, and its public health implications.
